# Prevalence of depression and anxiety in women with polycystic ovary syndrome (PCOS) and associated factors in a quaternary hospital in Thailand: a cross-sectional study

**DOI:** 10.1186/s12888-024-06154-8

**Published:** 2024-11-01

**Authors:** Pattra Keeratibharat, Areepan Sophonsritsuk, Ratana Saipanish, Penpun Wattanakrai, Makaramas Anantaburana, Siriluk Tantanavipas

**Affiliations:** 1 Department of Obstetrics and Gynaecology, Faculty of Medicine Ramathibodi Hospital, Mahidol University, Bangkok, 10400 Thailand; 2 Reproductive Endocrinology and Infertility Unit, Department of Obstetrics and Gynaecology, Faculty of Medicine Ramathibodi Hospital, Mahidol University, 270 Rama VI Road, Rajataewe, Bangkok, 10400 Thailand; 3 Department of Psychiatry, Faculty of Medicine Ramathibodi Hospital, Mahidol University, Bangkok, 10400 Thailand; 4 Division of Dermatology, Department of Medicine, Faculty of Medicine Ramathibodi Hospital, Mahidol University, Bangkok, 10400 Thailand

**Keywords:** Polycystic ovary syndrome, Depression, Anxiety, Mental well-being, Hirsutism

## Abstract

**Background:**

Polycystic ovary syndrome (PCOS) is a common gynaecological problem for women of reproductive age. Depression and anxiety are common conditions that occur in women with PCOS and have an impact on mental well-being. However, there is a lack of data on their prevalence and its associated factors in the Thai population. This cross-sectional study aimed to evaluate the prevalence of depression and anxiety among women with PCOS and identify the factors associated with depression and anxiety in women with PCOS as well as their impact on mental well-being in Thailand.

**Methods:**

A total of 260 women aged 15 to 40 years diagnosed with PCOS based on the Rotterdam criteria were included in the study. Physical examinations were conducted, and participants completed Hospital Anxiety and Depression Scale (HADS) and Thai version WHO-Five Well-Being Index (WHO-5 Thai) questionnaires to assess depression, anxiety, and mental well-being, respectively. The prevalence and prevalence rations (PR) with 95% confidence interval (CI) for depression and anxiety were analysed using modified Poisson regression analyses with robust variance estimators.

**Results:**

The prevalence of depression, anxiety, and poor mental well-being among women with PCOS was found to be 3.85%, 11.92%, and 16.92%, respectively. Abdominal obesity (PR 24.25, 95% CI: 2.75–219.50; *p* = 0.004), poor mental well-being (PR 16.68, 95% CI: 4.02–69.18; *p* = < 0.001), and snoring (PR 10.26, 95% CI: 2.06–51.14; *p* = 0.005) were identified as factors associated with depression in women with PCOS. Having children (PR 6.22, 95% CI: 2.90-13.35; *p* = < 0.001), alcohol drinking (PR 3.41, 95% CI: 1.52–7.65; *p* = 0.003), poor mental well-being (PR 2.32, 95% CI: 1.14–4.74; *p* = 0.021), and hirsutism (PR 2.23, 95% CI: 1.18–4.22; *p* = 0.014) were found to be relative factors for anxiety in women with PCOS.

**Conclusion:**

Women with PCOS is associated with high prevalences of depression and anxiety. Poor mental well-being was identified as key factors associated with both depression and anxiety in women with PCOS. Based on these findings, the present study suggests that screening for depression and anxiety should be conducted for all women with PCOS, especially those who present with poor mental well-being.

**Supplementary Information:**

The online version contains supplementary material available at 10.1186/s12888-024-06154-8.

## Background

Polycystic ovary syndrome (PCOS) is a common gynaecological condition in reproductive-aged women with a prevalence of 8–13% [1]. The clinical characteristics of PCOS are ovulatory dysfunction, hyperandrogenism, and polycystic ovaries on pelvic sonography [2]. PCOS is associated with infertility, obesity, insulin resistance (IR), diabetes mellitus (DM), and dyslipidaemia (DLP), which affect long-term complications, including reproductive, metabolic and psychological problems such as depression and anxiety [3–5]. Individuals with PCOS experience a higher prevalence of both depressive and anxiety disorders.

Depression, anxiety, and poor mental well-being are common mental health conditions. Depression is defined by a persistent low mood or loss of pleasure or interest in activities, while anxiety is characterised as excessive fear and worry, often accompanied by related behavioural disturbances. Additionally, mental well-being is an important component of overall quality of life (QoL). It is defined as a positive state of psychological, emotional, and social health, characterized by feelings of satisfaction, resilience, and the capacity to handle life’s difficulties effectively [6]. A recent meta-analysis reported a high prevalence of depression and anxiety (42% and 37%, respectively) in women with PCOS [3]. Furthermore, women with PCOS experienced a significantly decreased health-related QOL, including poor mental well-being [7]. Although the mechanism of association between mental health and PCOS is not fully understood, alterations in the metabolic pathway, including obesity and IR, and disturbances in the endocrine axes among women with PCOS may be potential pathophysiology for the emergence of depression and anxiety [4]. Negative body image resulting from clinical manifestations of hyperandrogenism, such as hirsutism, acne, and alopecia, contribute to a negative effect on mood and a reduction in self-esteem [8]. Moreover, women with PCOS who suffer from menstrual irregularity and/or infertility experience additional psychological stress and low QoL, which includes poor mental well-being [9]. A global study has revealed that the prevalence of depression, anxiety, and poor mental well-being is high in women with PCOS.

According to the latest estimates of cause-specific disability-adjusted life year (DALYs) for 2019, depression and anxiety are among the top 10 cause of DALYs in Thai women aged 20 to 40 years. They are reported as 530.06 and 517.62 DALYs per 100,000 population of depression and anxiety in Thai women aged 25 to 29 years, respectively [10]. However, these burdens tend to be lower than the global evidence, possibly influenced by cultural variations and ethnic backgrounds. The estimated prevalence of depression among Thai women is 3.5%, whereas the global prevalence is found to be 4.85%. Focusing on anxiety, its prevalence is approximately 5.35% among Thai women and 5.85% in the global population, respectively [11].

We hypothesise that prevalence of depression and anxiety among Thai women with PCOS, which may represent the Southeast Asia population, is lower than the global population with PCOS. Moreover, no study has been performed in the Thai population with that prevalence and its relative factors. Herein, the present study is designed to assess the prevalence of depression and anxiety in Thai women with PCOS to determine its associated factors and the impact of PCOS on mental well-being.

## Methods

### Study design

This cross-sectional study was conducted on women with PCOS who attended the Endocrine Clinic, Division of Reproductive Endocrinology & Infertility, Department of Obstetrics and Gynaecology, Faculty of Medicine Ramathibodi Hospital, Mahidol University, Bangkok, Thailand, a tertiary hospital centre, during the period from May 2021 to July 2022. The study was approved by the Ethical Clearance Committee on Human Rights Related to Research Involving Human Subjects, Faculty of Medicine Ramathibodi Hospital (MURA2021/290), and written informed consent was obtained from all participants. The study was reported using the Observational Routinely-collected Data (RECORD) guideline, as shown in Supplemental material 1.

### Participants

To determine the prevalence of depression and anxiety, previous studies by Cinar N at al [8] and Saima B et al. [12] were referenced. These studies used the formula for calculating sample size for estimating proportions in an infinite population. The sample sizes required to estimate the prevalence of depression and anxiety were determined to be 220 and 196, respectively, based on an expected proportion (P) of 0.173 for depression and 0.15 for anxiety, an alpha (α) of 0.05, and a standard normal variable (Z) of 1.96. (Supplemental material 2)

A total of 270 reproductive women aged 15 to 40 years, diagnosed with PCOS according to the 2003 Rotterdam criteria at our clinic during the study period, were initially considered for inclusion. The medical history of all clinic visitors was thoroughly reviewed using the medical record system. Following this review, 10 women were excluded based on the exclusion criteria, which included a history of mental disorders or the use of psychiatric medication prior to their PCOS diagnosis, the presence of coexisting chronic diseases that could impact mental health, such as cancer, recent severe stress events, such as the death of a family member or divorce within the past three months, and the inability to complete the questionnaire. Consequently, a total of 260 participants were included in the study.

All eligible patients were informed about the study’s purpose. For participants under the age of 18, both the individuals and their parents received an explanation of the study and signed the informed consent form. After obtaining informed consent, all eligible participants were referred to a private room for history taking, physical examination, and completion of the questionnaires.

### Data collection

#### Questionnaire

All participants answered a self-assessment questionnaire in the private room. The questionnaire consisted of 3 parts as follows:

1. A questionnaire addressing the sociodemographic and reproductive characteristics of women with PCOS includes sections on sociodemographic features (age, educational level, occupational status, monthly income, marital status, alcohol consumption, and smoking), medical history (self-assessment of the experience of snoring, comorbidities, current medications, history of hormonal treatment within the past three months, duration since PCOS diagnosis, and family history of PCOS or diabetes mellitus), and gynaecological history (age of menarche, parity, and fertility needs).
2. The Thai version of the Hospital Anxiety and Depression Scale (Thai HADS) questionnaire is a self-report rating scale composed of 14 questions, divided into 2 subscales. The first subscale, HAD-D, assesses symptoms of depression and consists of 7 items. The second subscale, HAD-A, assesses symptom of anxiety symptoms and consists of another 7 items. Each item is ranked on a scale of 0 (absence of symptoms) to 3 (maximum of symptoms). The total score for each subscale is ranged from 0 to 21. Depression and/or anxiety are defined as HAD-D and/or HAD-A scores ≥ 11. The sensitivity and specificity to detect depression are 85.71% and 91.35%, respectively. Sub-scales exhibit good internal consistencies with a Cronbach’s alpha of 0.8259. For anxiety measurement, HADs reveals 100% sensitivity and 86% specificity, with a Cronbach’s alpha coefficient of 0.8551 for the anxiety sub-scale [13, 14]. (Supplemental material 3)
3. The Thai version of the WHO-Five Well-Being Index (Thai WHO-5) questionnaire is used to measure current mental well-being and has adequate validity as a screening tool for depression. It is a self-rating inventory with 5 questions. Each statement, related to positive aspects of well-being, is rated from 0 (none of the time presenting symptoms) to 5 (all the time presenting symptoms), and the total score ranges from 0 to 25. The total score is multiplied by 4 to obtain a percentage final score, ranging from 0 (worst possible) to 100 (best possible mental well-being). Higher scores (> 50) on the Thai WHO-5 are defined as normal well-being, indicative of satisfactory well-being. A final score of ≤ 50% is reliable and valid to screen for major depression with a sensitivity of 89% and a specificity of 71% [15, 16]. (Supplemental material 4)

#### Anthropometric measurement

Blood pressure (BP), weight, height, waist circumference (WC), and hip circumference (HC) were examined by a single trained evaluator. A measure around the midpoint between the lowest palpable rib and the iliac crest was defined as WC. HC was measured at the widest part of the buttocks parallel to the floor [17]. WC divided by HC was calculated for the waist-hip ratio (WHR). Body mass index (BMI) was determined by dividing weight in kilograms by the square of height in metres. Hypertension (HTN) was defined as systolic blood pressure (SBP) ≥ 130 mmHg and/or diastolic blood pressure (DBP) ≥ 85 mmHg. Abdominal obesity was diagnosed when WC ≥ 80 cm. Overweight and obesity were indicated by BMI ≥ 23.0 to < 25 kg/m^2^ and ≥ 25.0 kg/m^2^, respectively.

#### Clinical features of PCOS

Irregular menstrual cycles as a sign of ovulatory dysfunction (OD), hyperandrogenism (HA), polycystic ovarian morphology (PCOM) assessed using ultrasonography, and acanthosis nigricans as a sign of IR were evaluated by a single trained investigator under the supervision of specialist physicians. Clinical features of PCOS were classified into 4 phenotypes: phenotype-A (OD + HA + PCOM), phenotype-B (OD + HA), phenotype-C (HA + PCOM), and phenotype-D (OD + PCOM) [18].

Irregular menstrual cycles were specified as shorter than 21 days or longer than 35 days of menstrual cycle or less than 8 cycles per year [19].

HA was defined as biochemical HA and/or clinical HA composed of acne, hirsutism, or alopecia. Acne was assessed according to the global acne grading system (GAGS) lesions of acne from 6 locations of the face and chest/upper back. The total score ranged from 0 to 44 and was classified into absent acne (score 0), mild acne (score 1–18), moderate acne (score 19–30), severe acne (score 31–38), and very severe acne (score > 38) [20]. Hirsutism was determined using the modified Ferriman-Gallway (mFG) score. Among Thai women, the number of hair follicles per unit of skin area is lower than that in black or white populations [21]. An mFG score of 3 or more indicated hirsutism [22], while moderate to severe hirsutism was determined by an mFG score ≥ 8. Alopecia was assessed by the Ludwig scale and interpreted into grades I, II, and III [23].

### Statistical analysis

Data analysis was conducted using Stata version 18. Descriptive statistics were reported as the mean and standard deviation (SD) or median and interquartile range (IQR) for continuous variables while frequency and percentages were used for categorical variables. The T test and chi-square test were employed for assessment of the normally distributed continuous and discrete data, respectively. Nonnormally distributed continuous data were analysed using the Mann‒Whitney U test. All variables were entered into univariate analysis, and those with a *p*-value < 0.1 were included in the multivariate analysis using modified Poisson regression analyses with robust variance estimators to examine factors associated with depression and anxiety in women with PCOS. Results are reported as prevalence ratios (PR) with 95% confidence interval (CI). A *p*-value < 0.05 was defined as statistically significant in all analyses.

## Results

### Demographic characteristics

Of 270 women screened, a total of 260 women met the qualified criteria and were recruited into the study. The mean age was 28.0 ± 4.9 years, and the age of menarche was 12.7 ± 1.6 years. Most of them were single (*n* = 210, 80.8%), childless (*n* = 250, 96.2%) and desired to have a child (*n* = 187, 71.9%). Two hundred and nine (80.4%) women were employed with a median monthly income of 540.5 [405.0,810.8] United Stated Dollars (USD). (Table 1)

**Table 1:** Sociodemographic and reproductive characteristics of women with PCOS classified by depression and anxiety

Characteristics	Total (*N* = 260)	Depression			Anxiety		
		Absence (*n* = 250)	Presence (*n* = 10)	*p* value	Absence (*n* = 229)	Presence (*n* = 31)	*p* value
Age (years)	28.0 ± 4.9	28.0 ± 4.9	25.6 ± 4.8	0.125	28.0 ± 4.7	27.6 ± 6.6	0.683
Age of menarche (years)	12.7 ± 1.6	12.7 ± 1.6	13.1 ± 2.0	0.544	12.6 ± 1.6	13.3 ± 1.8	0.032
Marital status							
- Single - Couple/married	210 (80.8) 50 (19.2)	201 (80.4) 49 (19.6)	9 (90.0) 1 (10.0)	0.692	188 (82.1) 41 (17.9)	22 (71.0) 9 (29.0)	0.140
Parity							
- 0 - ≥ 1	250 (96.2) 10 (3.8)	242 (96.8) 8 (3.2)	8 (80.0) 2 (20.0)	0.051	225 (98.3) 4 (1.7)	25 (80.6) 6 (19.4)	< 0.001
Fertility need	187 (71.9)	181 (72.4)	6 (60.0)	0.474	167 (72.9)	20 (64.5)	0.328
Educational level							
- High school or less - Bachelor or higher	24 (9.2) 236 (90.8)	23 (9.2) 227 (90.8)	1 (10.0) 9 (90.0)	1.000	20 (8.7) 209 (91.3)	4 (12.9) 27 (87.1)	0.504
Occupational status							
- Student - Unemployed - Employed	22 (8.5) 29 (11.1) 209 (80.4)	20 (8.0) 27 (10.8) 203 (81.2)	2 (20.0) 2 (20.0) 6 (60.0)	0.125	19 (8.3) 22 (9.6) 188 (82.1)	3 (9.7) 7 (22.6) 21 (67.7)	0.095
Monthly income in USD	540.5 [405.0,810.8]	540.5 [483.5,810.8]	337.8 [209.3,479.7]	0.006	540.5 [405.0,810.8]	405.5 [270.3,621.6]	0.009

**Abbreviations**: USD, United States Dollar

**Note**: Data are presented as the mean ± SD or median [25th, 75th percentile] or n (%) as appropriate

Table 2 includes the anthropometric and clinical features and disease profile of PCOS. The mean BMI, WC, HC, and WHR were 26.2 ± 6.4 kg/m^2^, 82.6 ± 14.9 cm, 99.3 ± 13.7 cm, and 0.83 ± 0.06, respectively. Overweight was observed in 20 (7.6%) women and obesity was found in 139 (53.5%) women. PCOS phenotype-A was the most common feature in the present study (*n* = 157, 60.4%). A median of 24.0 [12.0,48.0] months was the duration since PCOS diagnosis. Cyclic progestin and oral contraceptive pills (OCPs) were prescribed for the treatment of PCOS in 114 (43.8%) and 117 (45%) women, respectively. Only 29 (11.2%) women did not receive any hormonal treatment. The common comorbidity of PCOS was revealed in 67 (25.8%) women with DLP, 21 (8.1%) women with DM, 11 (4.2%) women with HTN, and 4 (1.5%) women with endometrial hyperplasia (EH). Moreover, 63 (24.2%) women suffered from snoring. Data from physical examination at the time questionnaires were administered, and 211 (81.2%) women had acne with a median acne score of 9.9 [2.0,16.8]. Hirsutism was demonstrated in 118 (45.4%) women with a median mFG score of 2.0 [1.0,5.0]. Most of them presented with mild hirsutism (*n* = 92, 35.4%). Forty-one (15.7%) women suffered from alopecia, and 36 women had mild degrees. Acanthosis nigricans was shown in 68 (26.2%) women.

**Table 2:** Anthropometric and clinical features and disease profile of PCOS classified by depression and anxiety

Characteristics	Total (*N* = 260)	Depression			Anxiety		
		Absent (*n* = 250)	Present (*n* = 10)	*p* value	Absent (*n* = 229)	Present (*n* = 31)	*p* value
BW (kg)	67.0 ± 17.6	66.5 ± 17.1	81.5 ± 23.8	0.008	66.4 ± 17.5	72.0 ± 17.7	0.103
BMI (kg/m^2^)	26.2 ± 6.4	26.0 ± 6.3	30.0 ± 7.2	0.054	26.0 ± 6.5	27.6 ± 5.8	0.188
- Overweight	20 (7.6)	20 (100)	0 (0)	1.000	16 (7.0)	4 (12.9)	0.274
- Obesity	139 (53.5)	131 (52.4)	8 (80)	0.111	118 (51.5)	21 (67.7)	0.089
WC (cm)	82.6 ± 14.9	82.0 ± 14.7	92.1 ± 16.6	0.039	82.1 ± 14.8	86.4 ± 14.6	0.132
HC (cm)	99.3 ± 13.7	98.0 ± 13.4	110.0 ± 15.8	0.011	98.9 ± 13.7	102.3 ± 13.3	0.185
WHR	0.83 ± 0.06	0.81 ± 0.06	0.83 ± 0.06	0.800	0.83 ± 0.06	0.84 ± 0.07	0.230
SBP (mmHg)	118.7 ± 13.1	118.5 ± 13.12	125.0 ± 11.7	0.116	118.5 ± 13.3	120.2 ± 12.0	0.500
DBP (mmHg)	77.7 ± 8.2	77.7 ± 8.2	79.4 ± 9.1	0.511	77.7 ± 8.1	77.8 ± 9.1	0.931
Acne - Mild - Moderate - Severe	211 (81.2) 160 (61.5) 41 (15.8) 10 (3.9)	201 (80.4) 152 (60.8) 39 (15.6) 10 (4.0)	10 (100.0) 8 (80.0) 2 (20.0) 0 (0)	0.216 0.413	185 (80.8) 137 (59.8) 39 (17.0) 9 (3.9)	26 (83.9) 23 (74.2) 2 (6.5) 1 (3.2)	0.680 0.392
Acne score	9.0 [2.0,16.8]	9.0 [2.0,16.3]	10.0 [8.0,19.5]	0.279	9.0 [2.0,17.0]	8.0 [3.0,13.0]	0.500
Hirsutism - Mild - Moderate to severe	118 (45.4) 92 (35.4) 26 (10.0)	113 (45.2) 90 (36.0) 23 (9.2)	5 (50.0) 2 (20.0) 3 (30.0)	0.754 0.106	99 (43.2) 77 (33.6) 22 (9.6)	19 (61.3) 15 (48.4) 4 (12.9)	0.042 0.165
mFG score	2.0 [1.0,5.0]	5.0 [1.0,5.0]	2.5 [4.3,9.0]	0.465	2.0 [0,5.0]	4.0 [1.0,5.0]	0.084
Alopecia - Mild - Moderate - Severe	41 (15.7) 36 (13.8) 4 (1.5) 1 (0.4)	38 (15.2) 34 (13.6) 3 (1.2) 1 (0.4)	3 (30.0) 2 (20.0) 1 (10.0) 0 (0)	0.197 0.160	35 (15.3) 30 (13.1) 4 (1.7) 1 (0.4)	6 (19.4) 6 (19.4) 0 (0) 0 (0)	0.599 0.688
Acanthosis nigricans	68 (26.2)	64 (25.6)	4 (40.0)	0.294	56 (24.5)	12 (38.7)	0.090
Irregular menstrual cycles	256 (98.5)	246 (98.4)	10 (10.0)	1.000	225 (98.3)	31 (100)	1.000
HA	206 (79.2)	197 (78.8)	9 (90.0)	0.693	178 (77.7)	28 (90.3)	0.105
PCOM	215 (82.7)	210 (84.0)	5 (50.0)	0.160	188 (82.1)	27 (12.6)	0.490
PCOS Phenotypes							
- A - B - C - D	157 (60.4) 46 (17.7) 4 (1.5) 53 (20.4)	153 (61.2) 41 (16.4) 4 (1.6) 52 (20.8)	4 (40.0) 5 (50.0) 0 (0) 1 (10.0)	0.081	133 (58.1) 42 (18.4) 4 (1.7) 50 (21.8)	24 (77.4) 4 (12.9) 0 (0) 3 (9.7)	0.242
Duration since diagnosis of PCOS (months)	24.0 [12.0,48.0]	24.0 [12.0,48.0]	40.5 [5.3,48.8]	0.719	24.0 [12.0,49.5]	19.0 [7.0,36.0]	0.233
Hormonal treatment							
- None - Cyclic P - OCP	29 (11.2) 117 (45) 114 (43.8)	28 (11.2) 109 (43.6) 113 (45.2)	1 (10.0) 8 (80.0) 1 (10.0)	0.052	24 (10.5) 102 (44.5) 103 (45.0)	5 (16.1) 15 (48.4) 11 (35.5)	0.489
Comorbidities							
- DM - HTN - DLP - EH	21 (8.1) 11 (4.2) 67 (25.8) 4 (1.5)	20 (8.0) 11 (5.4) 64 (25.6) 4 (1.6)	1 (10.0) 0 (0) 3 (30.0) 0 (0)	0.576 1.000 0.721 1.000	18 (7.9) 11 (4.8) 64 (27.9) 4 (1.7)	3 (9.7) 0 (0) 3 (9.7) 0 (0)	0.725 0.371 0.029 1.000
Family history							
- DM - PCOS	69 (26.5) 22 (8.5)	64 (25.6) 20 (8.0)	5 (50.0) 2 (20.0)	0.136 0.203	57 (24.9) 19 (8.3)	12 (38.7) 3 (9.7)	0.102 0.734
Snoring	63 (24.2)	56 (22.4)	7 (70.0)	0.002	52 (22.7)	11 (35.5)	0.119
Smoking	1 (0.4)	1 (0.4)	0 (0)	1.000	1 (0.4)	0 (0)	1.000
Alcohol drinking	5 (1.9)	5 (2.0)	0 (0)	1.000	3 (1.3)	2 (6.5)	0.110
Poor mental well-being	44 (16.9)	36 (14.0)	8 (80.0)	< 0.001	33 (14.4)	11 (35.5)	0.003

**Abbreviations**: BW, body weight; BMI, body mass index; WC, waist circumference; HC, hip circumference; WHR, waist-hip ratio; SBP, systolic blood pressure; DBP, diastolic blood pressure; mFG, modified Ferriman-Gallwey; HA, hyperandrogenism; PCOM, polycystic ovarian morphology; PCOS, polycystic ovary syndrome; Cyclic P, cyclic progestin; OCP, oral contraceptive pills; DM, diabetes mellitus; HTN, hypertension; DLP, dyslipidaemia; and EH, endometrial hyperplasia

**Note**: Data are presented as the mean ± SD or median [25th, 75th percentile] or n (%) as appropriate

### Prevalence of depression, anxiety, and poor mental well-being among women with PCOS

According to the Thai HADS questionnaire, the median [interquartile range, IQR] score for the assessment of depression was 4.0 [2.0,6.0] and the mean score for anxiety evaluation was 6.67 ± 3.14. The mean score of mental well-being assessed by the Thai WHO-5 questionnaire was 64.57 ± 14.13. The prevalence of depression and anxiety among women with PCOS assessed using the Thai HADS questionnaire was 3.85% and 11.92%, respectively. Poor mental well-being was detected in 16.92% by the Thai WHO-5 questionnaire. (Table 3).

**Table 3:** Prevalence of depression, anxiety disorders and poor mental well-being in 260 women with PCOS

Categories	*n* (%)
Depression	10 (3.85)
Anxiety	31 (11.92)
Poor mental well-being	44 (16.92)

A significantly higher prevalence of poor mental well-being was found in women with PCOS who had depression (80.0% vs. 14.0%, *p* = < 0.001) and anxiety (35.5% vs. 14.4%, *p* = 0.003). (Table 2).

In a comparison of the depression group, there was significantly higher body weight (81.5 vs. 66.5 kg, *p* = 0.008), WC (92.1 vs. 82.0 cm, *p* = 0.011), HC (110 vs. 98 cm, *p* = 0.01), and snoring (70% vs. 22.4%, *p* = 0.002) compared to those without depression. However, they had lower monthly income (337.8 vs. 540.5 USD, *p* = 0.006). (Tables 1 and 2)

Regarding anxiety, women with anxiety were found to have an earlier age of menarche (13.3 vs. 12.6 years, *p* = 0.032) and higher hirsutism prevalence (61.3% vs. 43.2%, *p* = 0.042) than those without anxiety. They also had a lower rate of being childless (80.6% vs. 98.3%, *p* = < 0.001), lower monthly income (405.4 vs. 5405 USD, *p* = 0.009), and lower comorbidity of DLP (9.7% vs. 27.9%, *p* = 0.029). (Tables 1 and 2)

### Factors associated with depression among women with PCOS

All statistically significant variables analysed by univariate binary logistic regression were entered into multivariate logistic regression. Abdominal obesity (prevalence ratios [PR] 24.25, 95% confidence interval [CI]: 2.75–219.50; *p* = 0.004), poor mental well-being (PR 16.68, 95% CI: 4.02–69.18; *p* = < 0.001), and snoring (PR 10.26, 95% CI: 2.06–51.14; *p* = 0.005) were demonstrated to be independent relative factors. (Table 4)

**Table 4:** Univariate and multivariate logistic regression analysis of depression in women with PCOS

Variables	Univariate			Multivariate		
	Prevalence ratios	95% CI	*p* value	Prevalence ratios	95% CI	*p* value
Parity ≥ 1	6.25	1.51–25.79	0.011			
WC ≥ 80 cm	8.21	1.05–64.10	0.045	24.55	2.75–219.50	0.004
SBP ≥ 130 mmHg	4.31	1.29–14.33	0.017			
DBP ≥ 85 mmHg	4.00	1.20-13.33	0.024			
Moderate or severe hirsutism (mFG score ≥ 8)	3.86	1.06–14.05	0.041			
Snoring	7.30	1.94–27.45	0.003	10.26	2.06–51.14	0.005
Anxiety	7.39	2.06–24.13	0.001			
Poor mental well-being	19.64	4.30-89.61	< 0.001	16.68	4.02–69.18	< 0.001

**Abbreviations**: WC, waist circumference; SBP, systolic blood pressure; DBP, diastolic blood pressure; mFG, modified Ferriman-Gallwey; CI, confidence interval

### Factors associated with anxiety among women with PCOS

After including all variables in univariate and multivariate analyses, using modified Poisson regression with robust variance estimators, the factors associated with anxiety in women with PCOS were having children (RR 6.22, 95% CI: 2.90-13.35; *p* = < 0.001), alcohol drinking (RR 3.41, 95% CI: 1.52–7.65; *p* = 0.003), poor mental well-being (RR 2.32, 95% CI: 1.14–4.74; *p* = 0.021), and hirsutism (RR 2.23, 95% CI: 1.18–4.22; *p* = 0.014). (Table 5).

**Table 5:** Univariate and multivariate modified Poisson regression analyses of anxiety in women with PCOS

Variables	Univariate			Multivariate		
	Prevalence ratios	95% CI	*p* value	Prevalence ratios	95% CI	*p* value
Parity ≥ 1	6.00	3.20-11.26	< 0.001	6.22	2.90-13.35	< 0.001
Employed	0.51	0.26–1.02	0.057			
Overweight and obesity (BMI ≥ 23 kg/m^2^)	2.65	1.12–6.24	0.026			
WHR	2.31	0.92–5.81	0.075			
Hirsutism (mFG score ≥ 3)	1.91	0.96–3.77	0.064	2.23	1.18–4.22	0.014
Acanthosis nigricans	1.78	0.91–3.48	0.090			
DLP	0.31	0.10-1.00	0.057			
Alcohol drinking	3.52	1.14–10.88	0.029	3.41	1.52–7.65	0.003
Depression	4.81	2.34–9.88	< 0.001			
Poor mental well-being	3.10	1.62–5.92	< 0.001	2.32	1.14–4.74	0.021

**Abbreviations**: WHR, waist-hip ratio; mFG, modified Ferriman-Gallwey; DLP, dyslipidaemia; CI, confidence interval

## Discussion

The present study focused on the adverse impact of PCOS on psychological impairment and worsened mental well-being. The prevalence of depression, anxiety, and poor mental well-being among women with PCOS from the present study was 3.85%, 11.92%, and 16.92%, respectively. Associated factors for depression in women with PCOS were abdominal obesity, poor mental well-being, and snoring. Moreover, having children, alcohol drinking, poor mental well-being, and hirsutism were related to women with anxiety in PCOS.

Comparing the results of the present study with the 2019 Global Burden of Disease (GBD) study in Thai women aged 15 to 39 years, the findings confirmed that the prevalence of depression (3.85% vs. 3.5%) and anxiety (11.92% vs. 5.35%) was higher in women with PCOS than in the general healthy Thai women population [11].

However, these prevalences reported in the present study were lower than those reported in previous studies. A meta-analysis conducted by Wang Y. et al. revealed that the prevalence of depression and anxiety in women with PCOS was 42% and 37%, respectively. When considering only the studies using the HADS questionnaire, it was observed that the prevalence of depression ranged from 30 to 46.5% and anxiety ranged from 17.3 to 63% [3]. Furthermore, the recent meta-analysis study conducted by Paweł D. et al. exhibited a 31% prevalence of depression measured with HADs [24]. According to the results from the 2019 GBD study, the study found that Thai women aged 15 to 39 have lower rates of depression (3.5% vs. 4.85%) and anxiety (5.35% vs. 5.85%) compared to global rates [11]. The findings from the present study were in line with these trends, suggesting that the low prevalence may be attributed to various factors, including differences in the study population, cultural variations, and ethnic backgrounds. Cultural perspective on body image dissatisfaction adversely impacts mental health.

The present study revealed that women with PCOS who experienced depression and/or anxiety had a significantly higher prevalence of poor mental well-being than those without PCOS (80.0% vs. 14.0%, *p* = < 0.001 and 35.5% vs. 14.4%, *p* = 0.003, respectively). These results aligned with various international studies reporting that women with PCOS who experienced depression and anxiety had significantly decreased QoL scores, regardless of the symptoms [25–27].

Additionally, poor mental well-being significantly correlated with both depression and anxiety among women with PCOS in the present study. The findings suggested a bidirectional relationship among depression, anxiety, and poor mental well-being, which could be attributed to various factors. These factors might include the somatic symptoms of PCOS, such as hirsutism, acne, and infertility, as well as the regulatory process related to depressive or anxiety symptoms. Consequently, the measurement of well-being informed treatment plans, aided in the development of effective interventions, and served as a protective factor against the onset or exacerbation of depression and anxiety.

Among women with PCOS who experienced depression, independent factors contributing to depression include not only poor mental well-being but also abdominal obesity and snoring. Abdominal obesity was identified as a key factor associated with depression in women with PCOS, consistent with findings from various studies [28, 29]. Abdominal obesity may affect plasma levels of adiponectin, leptin, cortisol, resistin, insulin, and inflammatory cytokines, such as tumour necrosis factor-α and interleukin-1β, which can enhance neuroinflammation and contribute to depression [30]. Whereas recent meta-analyses have reported a link between overall obesity and depression among women with PCOS [3, 4], our study did not find this association. Further research is needed to better understand the relationship among abdominal obesity, overall obesity and mental health outcomes in this population.

Focusing on snoring, sleep disturbance, including snoring, had a significant relationship with depression in Chinese women with an odds ratio (OR) of 1.34 (95% CI: 1.19–1.50) [31]. In women with PCOS, sleep disturbance and snoring, which are common symptoms of obstructive sleep apnoea, have been reported to have a higher prevalence [32] and exhibit a bidirectional relationship with depression [33, 34], which aligned with the findings of the present study.

Among women with PCOS who experienced anxiety, the present study found that having a child, hirsutism, and alcohol consumption were associated with anxiety, in addition to poor mental well-being, as previously mentioned.

Emphasizing having a child, these findings contrasted with previous studies that had shown a relationship between anxiety and infertility or not having a child [35]. However, various factors could induce anxiety. In the case of women with PCOS, the stressors related to parenting and the overall environment might have a stronger influence on anxiety than the symptoms of PCOS alone.

Hirsutism contributes to an undesirable body image, social avoidance, and low self-esteem and has a negative effect on mental health among women with PCOS [8, 36]. This condition has been identified as a major factor associated with anxiety, consistent with findings from several previous studies [37–39]. According to the treatment of hirsutism, Dokras A et al. revealed that a low-dose combined oral contraceptive pill combined with lifestyle modifications could reduce androgen levels and improve psychological well-being, including symptoms of anxiety [40]. However, the present study did not find a correlation between other clinical manifestations of hyperandrogenism, such as acne and alopecia, and depression and anxiety. These findings aligned with the International Evidence-based Guideline for the assessment and management of polycystic ovary syndrome 2023, which considered hirsutism as a single marker to predict biochemical hyperandrogenism in women with PCOS [2]. Although hirsutism was a significant associated factor, the impact of these symptoms on mental health varies among the individuals concerned and might be influenced by cultural and ethnic factors.

The identification of alcohol drinking as an associated factor for anxiety was in line with the current meta-analysis, which revealed a 1.59 times increased of alcohol consumption behaviour in early adults with anxiety (adjusted OR 1.59; CI 95% 1.22–2.07; *p* = 0.001) [41]. The in vitro study conducted by Congyue X et al. explained that alcohol-induced anxiety could occur via activating ferroptosis, which regulated synaptic plasticity post alcohol exposure [42].

For clinical implication, we suggest that depression and anxiety screening should be assessed in women with PCOS who have poor mental well-being. The strengths of the present study include being the first in Thailand to evaluate depression and anxiety in women with PCOS. One major strength is the adequate power of the sample size, which enhances the reliability and validity of the findings. Additionally, all aspects of PCOS related to mental health, such as body image (BMI, WHR, and WC), hyperandrogenism (hirsutism, acne, and alopecia), metabolic problems (DM, HTN, and DLP), and reproductive issues, were comprehensively evaluated.

There were some limitations in the present study. First, there was some selection bias and recall bias due to the cross-sectional methodology. Second, the use of reliable and valid questionnaires limited the full diagnosis of depression and anxiety. Third, biomarker-related PCOS, such as the free androgen index and homeostatic model assessment for IR, was not performed. Last, the present study was conducted in only one centre, which may limit generalizability. A multicentre study is recommended for further research to enhance the external validity of our findings.

## Conclusions

The prevalence of depression and anxiety in women with PCOS was 3.85% and 11.92%, respectively. These numbers were higher than those of the general population. Poor mental well-being are crucial independent associated factors for both depression and anxiety in women with PCOS. Other key factors associated with depression in PCOS include abdominal obesity and snoring, while factors related to anxiety are linked to negative body image due to hirsutism, as well as external factors such as having children and alcohol drinking. Consequently, we recommend that screening for depression and anxiety using the HADS questionnaire should be assessed in all women with PCOS, particularly for those exhibiting poor mental well-being. These screening can help identify individuals who may require additional support and interventions for their mental health.

## Electronic supplementary material

Below is the link to the electronic supplementary material.

Supplementary Material 1

Supplementary Material 2

Supplementary Material 3

Supplementary Material 4

## Data Availability

The datasets used and/or analysed during the current study are available from the corresponding author on reasonable request.

## Abbreviations

BMI: Body mass index
BP: Blood pressure
CI: Confidence interval
DBP: Diastolic blood pressure
DLP: Dyslipidaemia
DM: Diabetes mellitus
EH: Endometrial hyperplasia
GAGS: Global acne grading system
HA: Hyperandrogenism
HADS: Hospital Anxiety and Depression Scale
HC: Hip circumference
HTN: Hypertension
IQR: Interquartile range
IR: Insulin resistance
mFG: Modified Ferriman-Gallway
OCP: Oral contraceptive pills
OD: Ovulatory dysfunction
OR: Odds ratio
PCOM: Polycystic ovarian morphology
PCOS: Polycystic ovary syndrome
PR: Prevalence rations
QoL: Quality of life
SBP: Systolic blood pressure
SD: Standard deviation
USD: United Stated dollar
WC: Waist circumference
WHO-5 Thai: Thai version of the WHO-Five Well-Being Index
WHR: Waist-hip ratio
