# Supplementary material for: Prevalence of depression and anxiety in women with polycystic ovary syndrome (PCOS) and associated factors in a quaternary hospital in Thailand: a cross-sectional study
Source: BMC Psychiatry. 2024 Nov 1;24:760. doi: 10.1186/s12888-024-06154-8 (PMC11529037; doi:10.1186/s12888-024-06154-8)
Supplement: Supplementary file 2 — Supplementary Material 2 [file 12888_2024_6154_MOESM2_ESM.docx]

**Supplemental material 2: Sample size calculation**

These studies used the formula for calculating sample size for estimating proportions in an infinite population. Based on the studies demonstrated by Cinar N at al. [1] and Saima B et al. [2], they revealed that the prevalence of depression and anxiety in women with PCOS were 17.3% and 15%, respectively. These results were entered to the infinite population proportion formula;

$$n = \frac{Z_{\alpha/2}^{2}P(1-P)}{e^{2}}$$

The sample size for determined prevalence of depression

Alpha (α) = Significance level 0.05

Z (α/2) = 1.96 for 95% CI

Proportion (P) = 0.173

Error (d) = 0.05

Sample size = 220

The sample size for determined prevalence of anxiety

Alpha (α) = Significance level 0.05

Z (α/2) = 1.96 for 95% CI

Proportion (P) = 0.15

Error (d) = 0.05

Sample size = 196

At an expected proportion (P) of 0.05, an alpha (α) of 0.05, and a standard normal variable (Z) of 1.96, the sample size for determined the prevalence of depression and anxiety was 220 and 196, respectively. The total of 260 reproductive women were included in this study.

**References**

1. Cinar N, Kizilarslanoglu MC, Harmanci A, Aksoy DY, Bozdag G, Demir B, et al. Depression, anxiety and cardiometabolic risk in polycystic ovary syndrome. Hum Reprod. 2011;26(12):3339-45.

2. Batool S, Ahmed F, Ambreen A, Sheikh A, Faryad N. Depression and anxiety in women with polycystic ovary syndrome and its biochemical associates. SAFOG. 2016;8(1):44-7.
