# Supplementary material for: Prevalence of depression and anxiety in women with polycystic ovary syndrome (PCOS) and associated factors in a quaternary hospital in Thailand: a cross-sectional study
Source: BMC Psychiatry. 2024 Nov 1;24:760. doi: 10.1186/s12888-024-06154-8 (PMC11529037; doi:10.1186/s12888-024-06154-8)
Supplement: Supplementary file 4 — Supplementary Material 4 [file 12888_2024_6154_MOESM4_ESM.docx]

**Supplemental material 4: The WHO-Five Well-Being Index (WHO-5) and the Thai version of the WHO-Five Well-Being Index (Thai WHO-5) questionnaire**

**
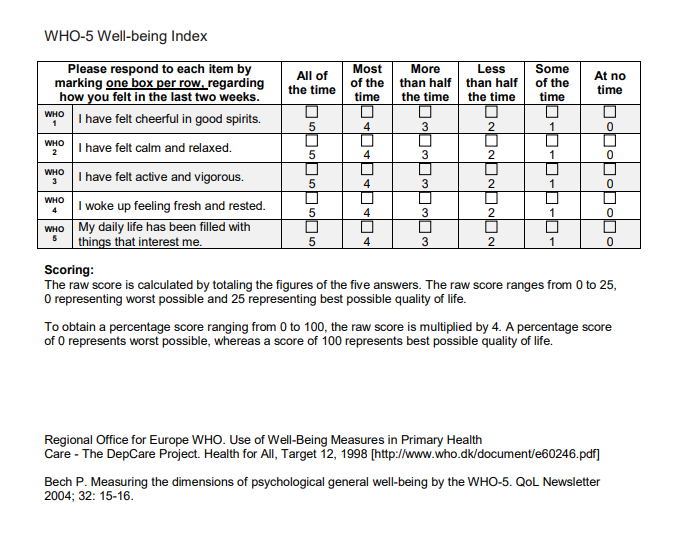
**
